# Intrinsic Electronic Structure and Inhomogeneity of High-Entropy Layered REOBiS_2_ Superconductors

**DOI:** 10.1021/acs.inorgchem.5c01680

**Published:** 2025-05-29

**Authors:** F. Minati, G. Tomassucci, M. Hattori, Y. Fujita, M. Nagao, L. Tortora, A. Barinov, M. Kopciuszynski, G. Campi, L. Boeri, T. Mizokawa, N. L. Saini

**Affiliations:** † Dipartimento di Fisica, 9311 Università di Roma “La Sapienza”, P. Aldo Moro 2, 00185 Roma, Italy; ‡ Department of Applied Physics, 13148 Waseda University, Shinjuku, 169-8555 Tokyo, Japan; § Center for Crystal Science and Technology, 38146 University of Yamanashi, Kofu, Yamanashi 400-0021, Japan; ∥ Sincrotrone Trieste S.C.p.A., Area Science Park, 34012 Basovizza, Trieste, Italy; ⊥ Institute of Crystallography, CNR, via Salaria Km 29.300, I-00015 Monterotondo, Roma, Italy

## Abstract

Two decades after the discovery of high-entropy alloys (HEAs), the field has witnessed these systems rise as prominent examples of high-performance functional materials, overcoming established knowledge of multicomponent systems. HEA superconductors are currently under thorough investigation due to their robust superconducting state and the possibility of enhancing their figure of merit through the high-entropy approach, in addition to the well-known mechanical and thermal properties of these materials. Here, we have investigated the electronic structure of HEA-type REOBiS_2_ layered superconductors (RE = rare earth) using spectromicroscopy and angle-resolved photoemission spectroscopy (ARPES) with a submicron beam size. The overall features of the fundamental electronic structure are robust, showing limited effects of mixing entropy. We find an inherent coexistence of phases driven by local fluctuations in the interlayer interactions. This coexistence exhibits distinct patterns for different samples characterized by varying configurational entropy. Similarly, the Luttinger volume estimated from the ARPES spectra reveals differing self-doping regimes, indicating that RE valence fluctuations are possibly influenced by configurational disorder. Overall, this study represents the first report on the electronic structure of HEA-type BiS_2_-based superconductors and provides valuable insight into controlling superconducting properties by tailoring nano- to microstructures through a high-entropy approach.

## Graphical abstract

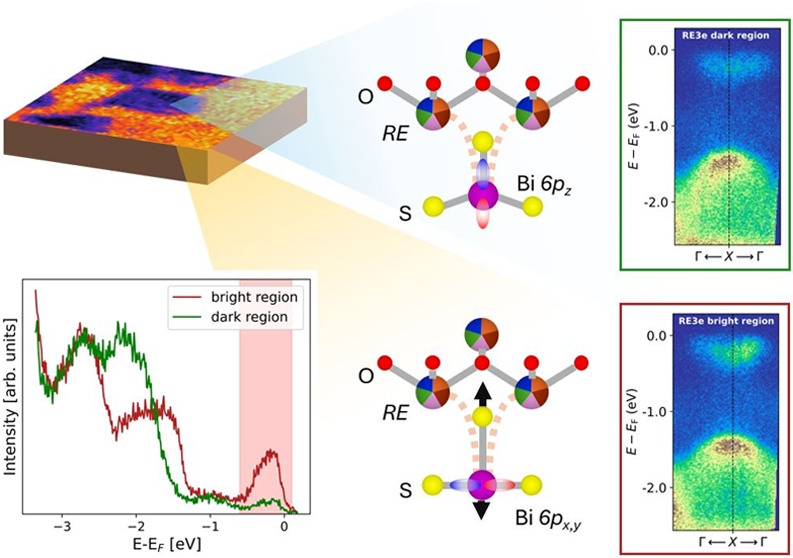

## Introduction

High-performance materials constitute the backbone of modern technological applications, where ever-increasing advanced functionalities are needed to address complex challenges in key fields, such as energy, aerospace, electronics, and healthcare. In the last two decades, multicomponent materials or high-entropy alloys (HEAs) have ubiquitously emerged as a forefront paradigm in the design of high-performance materials due to their extraordinary functional properties.^1^ The HEA solid solutions are favored by the high configurational mixing entropy, defined as Δ*S*_mix_ = −*R*∑_i_ *c*_i_ln*c*_i_, where *R* and *c*_i_ are the universal gas constants and the compositional ratio. The countless unexpected properties and the almost unlimited compositional space hold the latent potential to deeply affect future materials development; however, a coherent understanding of the relationship between composition, microstructure, and physical properties is still missing.

In this framework, HEA superconductors represent one of the hot topics^2^ owing to the reported enhancement of superconducting properties^3,4^ and the outstanding robustness of the superconducting phase against extremely high pressures,^5,6^ magnetic fields,^7,8^ and irradiation.^9,10^ Among them, layered HEA superconductors have the significant advantage of having the high-entropy layers separately stacked with the superconducting layers, limiting the direct effect of disorder on the superconducting state. BiS_2_-based layered superconductors are a good example of such systems, given the remarkable correlation between structural and electronic properties prompted by a peculiar instability of the BiS_2_ lattice hosting the superconductivity^11–13^ and by the local configuration of the overlying REO layer^14^ (Figure 1). Furthermore, the structural and electronic behavior of the BiS_2_ superconducting layer is largely affected by an intrinsic in-plane atomic disorder, as it has been revealed by means of experimental probes sensitive to the local structure.^15–17^

**Figure 1:**
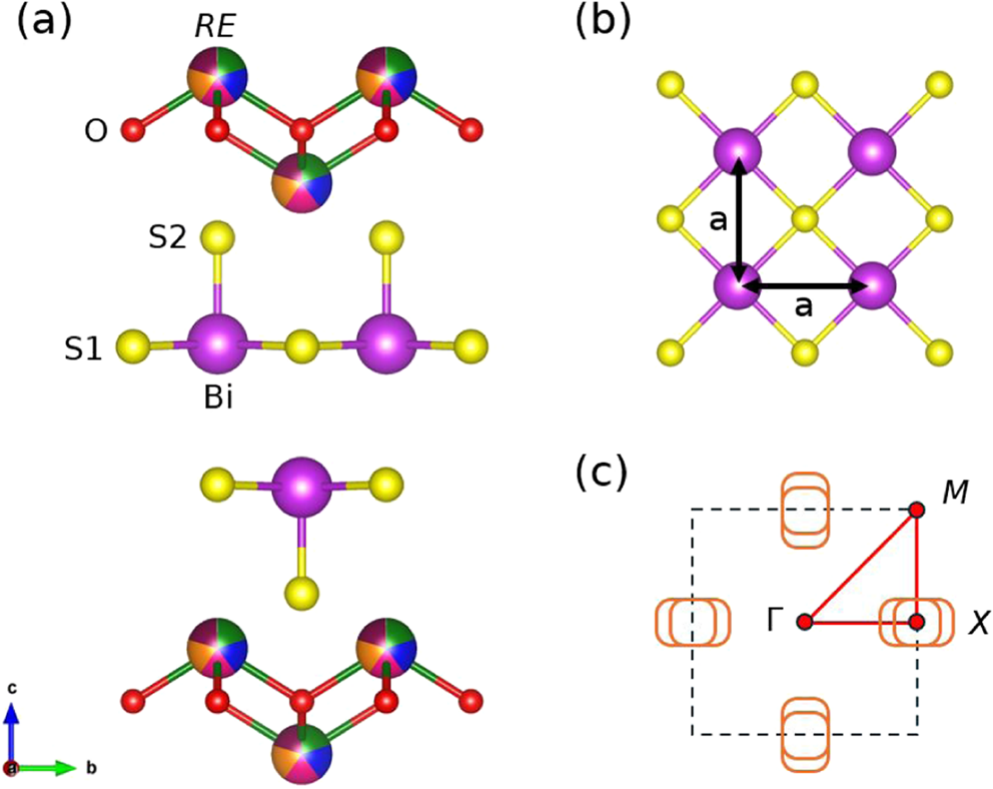
(a) Crystal structure of REOBiS_2_ for a generic high-entropy RE site. Nonequivalent in-plane and out-of-plane S sites are indicated by numbers S1 and S2. (b) Top view of the ideal square Bi–S1 plane. The BiS_2_ plane is expected to undergo severe distortions due to the Bi 6s lone pair. (c) Schematic depiction of the typical Fermi surface of doped BiS_2_ systems. The Brillouin zone is shown by dashed squares, and high symmetry directions are indicated by red solid lines.

The parent phase of the REOBiS_2_ family of compounds is semiconducting, and metallicity can be induced by substitution of F^–^ for O^2–^ or RE^4+^ in place of RE^3+^.^18,19^ Given the two-dimensional nature of the active layer, the doped electrons give rise to an electron pocket with Bi 6p_*x*,*y*_ character around the *X-*point (Figure 1c) of the square Brillouin zone.^20–22^ In addition, due to high structural susceptibility, local optimization of the BiS_2_ lattice can be achieved through chemical pressure either by Se substitution for S in the BiS_2_ layer or by RE substitution in the REO blocking layer.^23,24^ This route provides a way to increase the superconducting critical temperature *T*_c_, which typically ranges from 2 to 11 K in F-doped REOBiS_2_ systems.^25^ In particular, it has been found that an enhanced *T*_c_ for REO_0.5_F_0.5_BiS_2_ compounds is achieved by increasing the atomic number of the RE element.^26^ The higher the RE atomic number, the smaller the atomic radius, producing a shrinkage of the crystal unit cell along the *a*-axis. This compression shrinks the adjacent BiS_2_ active layer as well, causing an increase in *T*_c_. Therefore, it is possible to tune the structure and degree of chemical pressure and thus change the superconducting properties of these materials by changing the RE site.^27^

In undoped REOBiS_2_ superconductors, the RE site determines the superconducting transition temperature. The RE site of REOBiS_2_ superconductors can be substituted for various RE elements, such as La/Ce,^28^ Ce/Nd,^29^ and Ce/Pr.^30^ Indeed, superconductivity can also be achieved by exploiting valence fluctuation at the RE site, as already reported for self-doped CeOBiS_2_ and EuFBiS_2_ superconductors.^31–33^ Recently, several BiS_2_-based superconductors have been synthesized with a HEA-type REO blocking layer, where the RE site is randomly occupied by several rare-earth species.^34^ By investigating the high-entropy effects in the optimally doped REO_0.5_F_0.5_BiS_2_ compounds, a clear relation between the increasing configurational entropy at the RE site and the reported improvements of the superconducting properties emerges, associated with a suppression of the planar disorder of the adjacent conducting layer.^3^ Nevertheless, extrinsic doping by substitution also affects the disorder. Therefore, self-doping is more appropriate to explore the effect of high entropy.

In this work, we aim to address the issue of a possible relationship between the high configurational entropy and the electronic properties of this novel class of high-entropy superconductors. We have investigated the electronic structure of HEA-type REOBiS_2_ single crystals by scanning photoemission (SPEM) and angle-resolved photoemission spectroscopy (ARPES) using submicron size beam. This novel experimental approach has critical importance in the study of inhomogeneous solid-state systems, since it allows us to directly measure the realistic electronic structure, exploiting a submicrometer space resolution. In particular, we have analyzed two samples with different RE equivalent substitutions: Ce_0.33_Pr_0.33_Nd_0.33_OBiS_2_ (RE3e) and La_0.20_Ce_0.20_Pr_0.20_Nd_0.20_Sm_0.20_OBiS_2_ (RE5e), characterized by mixing entropy Δ*S*_mix_ = 1.098 and 1.598 *R*, respectively (*R* being the universal gas constant). The space-resolved photoemission reveals phase separation in distinct electronic phases, characterized by different densities of occupied states in the conduction band. They are well separated in space on a mesoscopic length scale, showing the typical electron pocket at the *X-*point of the Brillouin zone found in BiS_2_-based superconductors.^35,36^ The results have direct implications on the understanding of how the microstructure can affect the electronic properties of HEA-type superconductors, and in turn, it opens plausible ways to tailor new HEA-type superconductors by manipulating the atomic correlations through the high-entropy approach.

## Results and Discussion

Figure 2(a,b) shows SPEM maps of RE3e and RE5e single crystal samples at 100 K, measured right after the in situ cleaving in the ultrahigh vacuum measurement chamber. The color scale represents the integrated intensity of the photoemission spectrum in the energy range −3.0 eV ≤ *E* – *E*_F_ ≤ 0.17 eV, probing the electronic states at the *X*-point of the Brillouin zone (Figure 1c), where we expect to find possible bands crossing the Fermi energy. In the selected energy interval, the spectral weight of Bi 6p_*x*,*y*_, S 3p, and admixed RE 4f contributions are expected. Within the spatial resolution (1 × 1 μm^2^ for RE3e and 2 × 2 μm^2^ for RE5e), the probed areas are seemingly homogeneous and flat, albeit with small morphological defects and steps due to a nonperfect cleaving. Instead, in the vicinity of the Fermi level (−0.6 eV ≤ *E* – *E*_F_ ≤ 0.1 eV), probing a spectral weight of Bi 6p_*x*,*y*_ states, a strong contrast becomes clearly visible (Figure 3(a,c)), indicating phase separation in both samples featuring different electronic structures. In particular, bright domains highlight an enhanced metallic phase with a larger spectral weight due to Bi 6p_*x*,*y*_ states near *E*_F_. This observation suggests that the high-entropy REOBiS_2_ is electronically inhomogeneous with coexisting phases, similar to pristine EuFBiS_2_ and CeOBiS_2_,^37,38^ whose hallmark is a different density of states in the conduction band. The RE5e system sparsely exhibits such inhomogeneities with domains ranging from roughly 10–20 μm, while in RE3e, these domains are much larger with a dimension exceeding 50 μm. We can see that such patterns are randomly distributed all over the surfaces of the two samples, exhibiting hardly any spatial correlation with sample edges or defects. Therefore, the physical nature of the inhomogeneities in high-entropy REOBiS_2_ bears more similarities with self-doped EuFBiS_2_ rather than the stoichiometric CeOBiS_2_, where the metallic phase is believed to be induced by strain effects near the structural defects.^37,38^ In addition, the domains are extended to mesoscale, characteristic of the EuFBiS_2_ system.

**Figure 2:**
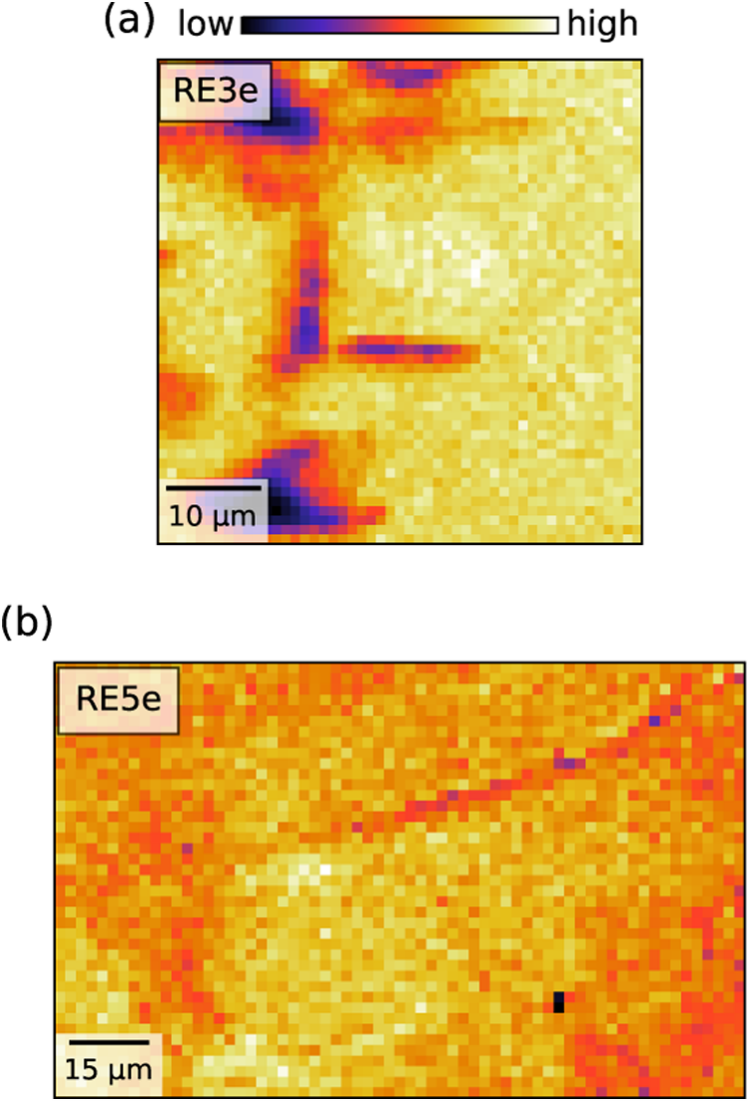
Scanning photoelectron microscopy (SPEM) maps of RE3e (upper panel) and RE5e (lower panel) crystals measured at 100 K using an incident photon energy of *hν* = 27 eV. The maps were acquired by setting the photoelectron analyzer to probe the X-point of the BZ. In particular, part (a) shows a SPEM image of a 50 × 50 μm^2^ area of RE3e exploiting a spatial resolution of 1 × 1 μm^2^, while part (b) is a 128 × 80 μm^2^ SPEM image of RE5e with a 2 × 2 μm^2^ spatial resolution. In both images, each pixel intensity is obtained by integrating the photoemission spectrum within – 3.0 eV ≤ *E* – *E*_F_ ≤ 0.17 eV.

**Figure 3:**
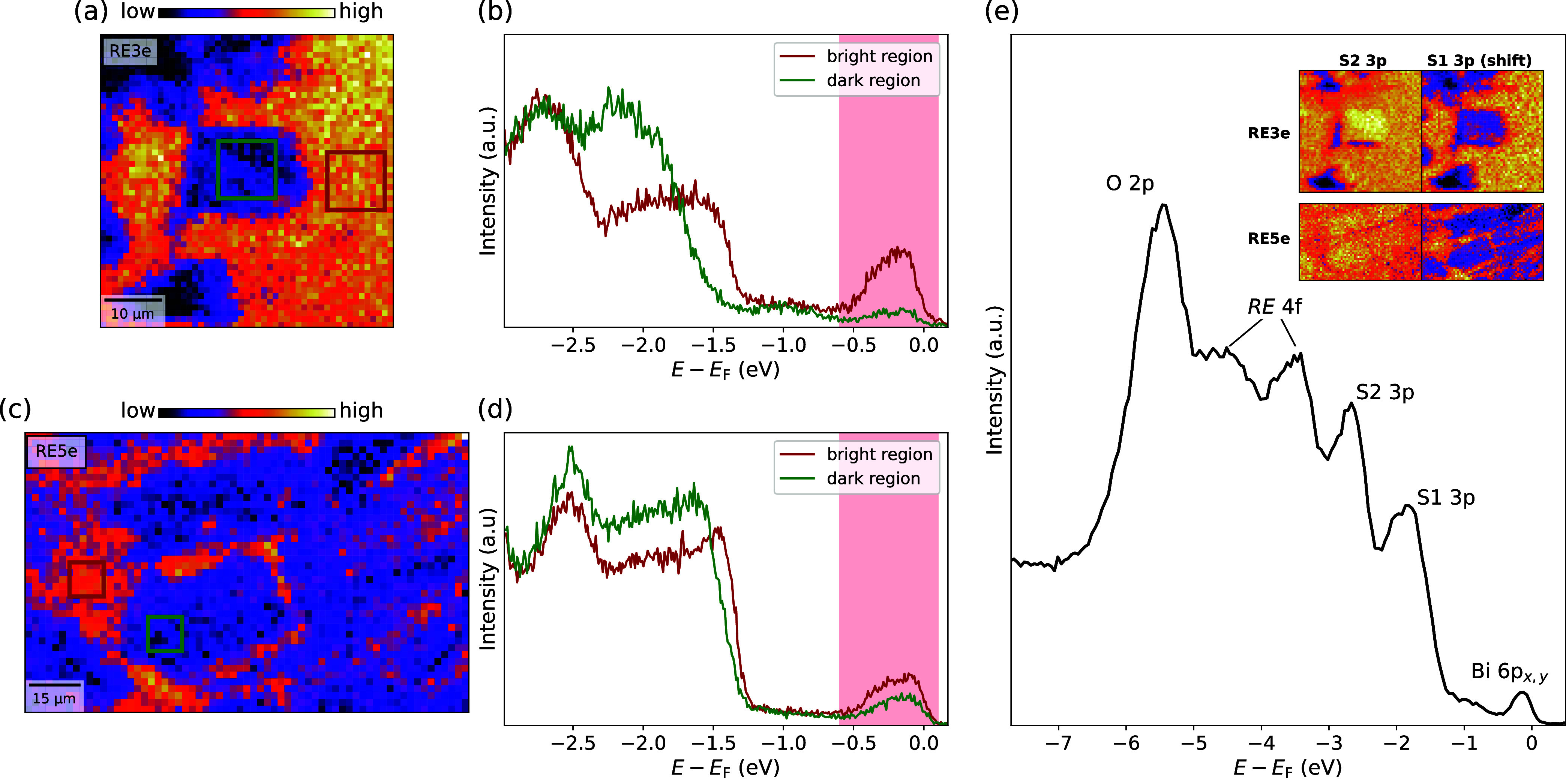
SPEM images (left) of RE3e (a) and RE5e (c) in the same spatial region and angle of the analyzer as the ones in Figure 2 but integrating in a different energy range of −0.6 eV ≤ *E* – *E*_F_ ≤ 0.1 eV. (b–d) Photoemission spectra integrated over a small spatial region of 10 × 10 μm^2^ outlined by green and red squares in the SPEM images (a) and (c). The highlighted red areas indicate the energy range where the SPEM maps shown in parts (a) and (c) have been integrated. (e) Angle-integrated photoemission spectrum of RE3e measured with a beam size of 500 × 500 nm^2^ within the bright region. The inset shows SPEM maps of RE3e and RE5e integrated over the energy range of the S2 3p peak and over the shifted energy region of peak S1 3p, clearly visible in parts (b) and (d).

Figure 3(b,d) represents the local photoemission spectra integrated over small regions of 10 × 10 μm^2^ highlighted in the corresponding SPEM images of RE3e and RE5e (Figure 3(a,c)). We can notice two major changes in both samples driving the space evolution between the two phases: (i) the bright regions seem to have enhanced spectral weight associated with the conduction band and (ii) a distinct suppression of the peak at −2 eV, which also suffers an energy shift of ∼0.1–0.2 eV toward the Fermi level. The features related to the enhanced metallic phase are more pronounced in RE3e in comparison to RE5e. This is likely due to an optimized charge transfer mechanism (discussed later). Figure 3(e) shows the typical angle-integrated valence band spectrum, allowing us to identify the relevant contributing electronic orbitals near the Fermi level. In particular, we can see that the increased number of occupied states in Bi 6p_*x*,*y*_ orbitals stems from an evolution of the local structure of the BiS_2_ lattice, which manifests itself through a lower binding energy of the S1 3p orbitals. Furthermore, this correlation is clearly present all over the surface of both samples, as we can apparently observe in the inset of Figure 3(e). In fact, integrating over an energy range corresponding to the shifted shoulder of the S1 3p peak (approximately −1.5 eV ≤ *E* – *E*_F_ ≤ −1.2 eV), the photoemission maps of both samples display exactly the same pattern as in Figure 3(a,c). In addition, the S2 3p intensity appears to have an anticorrelation with the enhanced metallic phase.

The nature of the spatial evolution can be further disentangled from the band dispersion probed by ARPES measurements performed by using a nanofocused beam (500 × 500 nm^2^). Fermi surfaces have been obtained at different points on both samples, and they always feature the same geometry consisting of rectangular electron pockets around the *X-*points of the square Brillouin zone. Figure 4(c) illustrates the characteristic Fermi surface of RE3e and RE5e, obtained by symmetrization of the integrated spectral intensity within an energy window *E*_F_ ± 0.1 eV. The measured Brillouin zone exhibits the same features as the typical Fermi surface introduced in Figure 1(c) and measured in several other BiS_2_-based superconductors.^13,20,36,37^ This merely indicates that the high-entropy REOBiS_2_ conduction band mostly originates from Bi 6p_*x*,*y*_ orbitals. Similarly, the band structure of both samples in the *M*-Γ-*X* high symmetry directions appears equivalent to other pristine BiS_2_-based superconductors, at least in the considered energy range −2.5 eV ≤ *E* – *E*_F_ ≤ 0.2 eV. Dispersing Bi 6p_*x*,*y*_ bands crossing the Fermi level can be clearly identified in Figure 4(a,b). In both figures, the left and right panels show the band structure measured within the bright and dark regions of Figure 3. The difference of Bi 6p_*x*,*y*_ spectral weight between the two phases is more evident in the RE3e sample than in RE5e.

**Figure 4:**
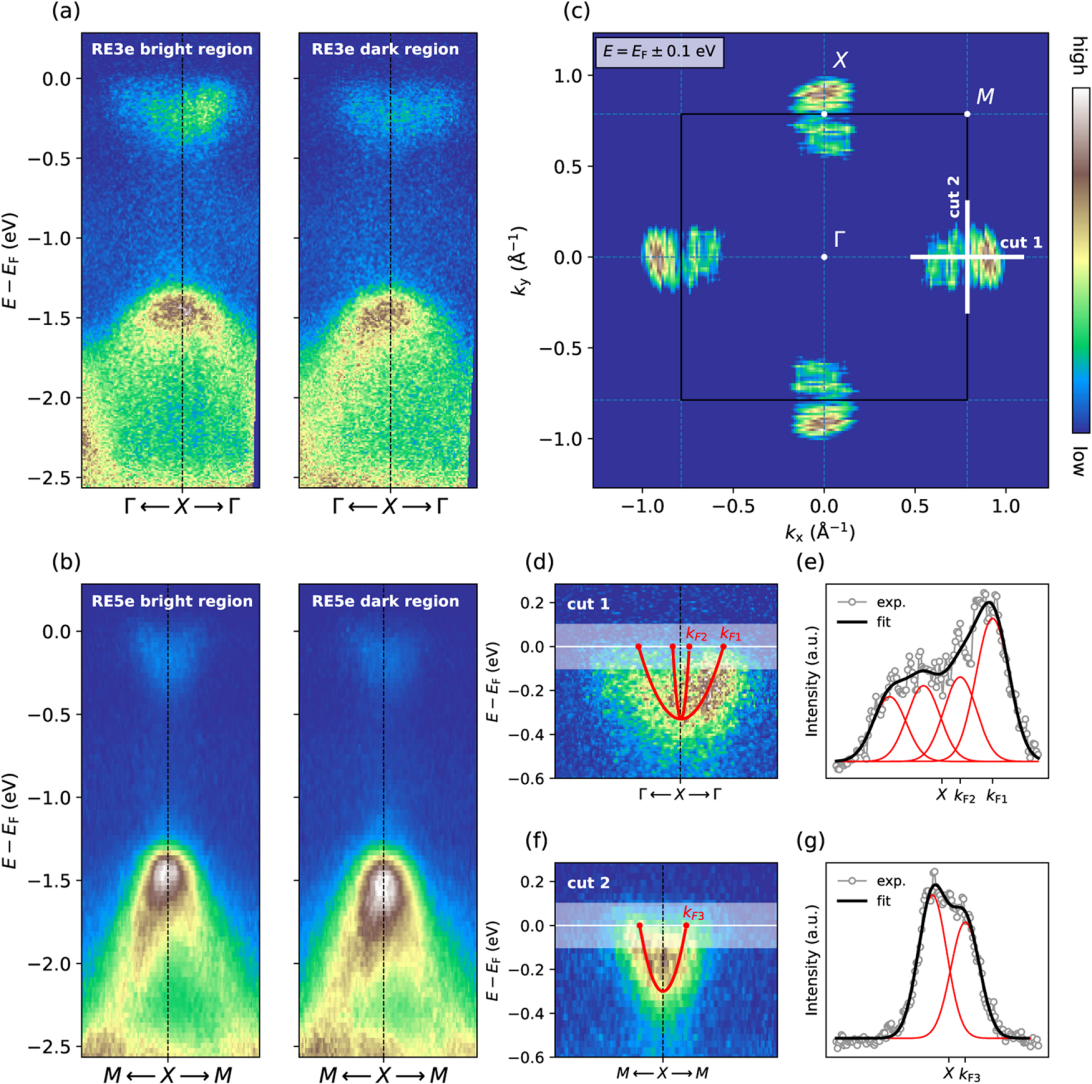
(a,b) Space-resolved ARPES spectra acquired within bright (left) and dark (right) regions using a submicron size (500 × 500 nm^2^) beam and photon energy of 27 eV. (a) Band dispersions of RE3e along the high-symmetry direction Γ – *X* – Γ, while in part (b), RE5e band dispersion is displayed along the *M* – *X* – *M* direction. (c) Symmetrized Fermi surface map integrated over *E*_F_ ± 0.1 eV for RE3e and RE5e. (d–f) Details of the conduction band dispersions along cut 1 and cut 2 (see part (c)). Superimposed parabolic bands have been obtained by estimating the bottom of the band through the energy distribution curve and the Fermi wave vectors by means of the fitting process shown in parts (e) and (g). The experimental momentum distribution curves integrated in *E*_F_ ± 0.1 eV have been fitted using four (cut 1) and two Gaussians (cut 2).

One of the highly debated issues in BiS_2_-based superconductors has been the discrepancy between the nominal doping and the actual number of electrons estimated from the size of the Fermi surface.^35,39,40^ In order to explore the behavior of high-entropy REOBiS_2_, we estimated the size of the Fermi surface belonging to enhanced metallic regions of both RE3e and RE5e samples. In particular, we have extracted the momentum distribution curves (MDCs) along two cuts denoted as cuts 1 and 2 in Figure 4(c) and integrated them over the energy range *E*_F_ ± 0.1 eV, as depicted in Figure 4(d,f). The obtained profiles have been extrapolated employing two Gaussian functions along the *M* – *X* – *M* direction and, due to the predicted band splitting,^21^ four Gaussians along the Γ – *X* – Γ direction. The fitted curves and integrated experimental MDCs are shown in Figure 4(e,g). From the fitted peak positions, we have directly retrieved the Fermi wave vectors denoted as *k*_F1_, *k*_F2_, and *k*_F3_. Then, exploiting the Luttinger theorem, we estimated the effective carrier doping, obtaining *x*_3*e*_ = 0.108 ± 0.007 e^–^/unit cell and *x*_5*e*_ = 0.083 ± 0.007 e^–^/unit cell.

Here, the amount of self-doped carriers is supposed to be provided by the valence fluctuations of Ce^3+^/Ce^4+^ and Pr^3+^/Pr^4+^, while other RE species are less likely to present this instability in their ground state. This has been also shown by X-ray absorption measurements.^41^ In addition, considering that the effect of electron correlations is expected to be negligible, a possible charge transfer mechanism based on local structure has been proposed.^14,42–44^ In particular, it has been shown that different phases arise as a result of different structural configurations, represented in Figure 5, which either promote or inhibit the transfer of self-doped electrons in the REO layer to the conductive BiS_2_ layer. Local distortions driven by the instability of the BiS_2_ layer can lead to a shortening of the Bi–S2 distance, enabling unoccupied Bi 6p*_z_* states to capture extra RE 4f electrons. The random arrangement in real space of such localized electrons, originating from Bi–S planar disorder, makes these states to appear as a nondispersing feature around −1 eV in ARPES spectra.^32^ On the other hand, the local configuration illustrated on the right-hand side in Figure 5 is expected to assist the charge transfer RE–S2-Bi pathway, which allows self-doped electrons to reach the Bi 6p_*x*,*y*_ states near *E*_F_, namely improving the metallic character. In this case, the role of S2 is vital, as shown by local structure studies^33^ and density functional theory (DFT) calculations^37^ for self-doped systems.

**Figure 5:**
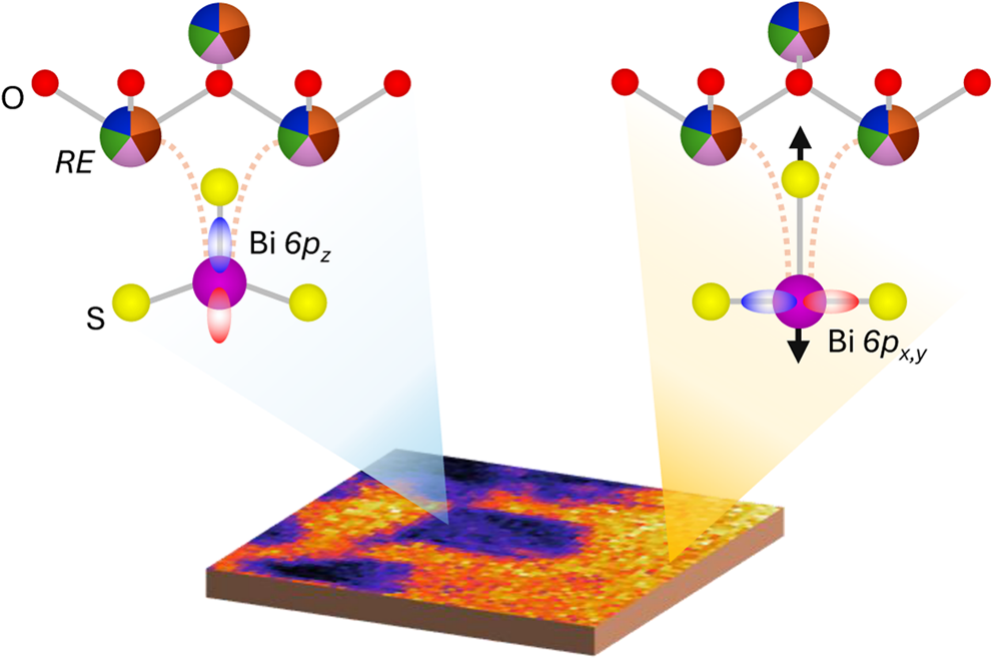
Possible local structures give rise to different phases. The configuration on the right side is expected to enhance the metallic character of BiS_2_-based systems.

As the characteristic disorder in the BiS_2_ layer is reported to be suppressed in the high-entropy RE­(O,F)­BiS_2_,^3^ we expect the differences in the estimated Luttinger volumes are direct consequences of a different RE^3+^/RE^4+^ ratio. Nonetheless, the local distortions are likely responsible for the reported phase separation, as depicted in Figure 5. This explanation is not only consistent with analogous reports on different BiS_2_-based superconductors,^37,38^ but it also accounts for the major spectroscopic features characterizing the two phases, namely, the enhanced Bi 6p_*x*,*y*_ contribution and the suppression of the spectral weight of S1 3p associated with a noticeable shift toward lower binding energy. The higher relative intensity of such features, highlighted for RE3e, can be associated with a smaller mean RE atomic radius compared to that in RE5e. Indeed, smaller RE size is known to enhance the metallic phase associated with the underlying structural configuration.^14^

Besides the above, it is noticeable that in the inherent microstructure of the phase separation in the parent CeOBiS_2_, the metallic phase emerges only in correspondence with morphological defects where the local strain makes the metallic configuration energetically favorable, while in the HEA counterparts, the phase separation is not accidental and it brings out different characteristic length scales in different samples. This phase coexistence cannot be overlooked, as it has proved to have primary importance in several superconducting systems, such as high-*T*_c_ superconductors^45–48^ and other high-entropy superconductors.^49^ As we ruled out chemical inhomogeneities underlying the two phases, their intrinsic concomitance is likely to be a consequence of nanoscale electron texturing. In this regard, experimental evidence of local ordering in HEAs^50,51^ recently prompted a novel understanding of short-range ordering and its formation mechanism in multicomponent systems.^52,53^ The effort of building a scheme beyond the picture of the random solid solution also unveiled how incipient long-range ordering emerges in HEAs, especially at low temperatures.^54,55^ Therefore, the observed differences in the two phase separation patterns, and in turn, their superconducting properties,^56^ may arise as a result of distinct short-range orderings dictated by the quite different substitutions in RE3e and RE5e samples. A comprehensive relation between substitution strategies, microstructure, and physical properties is still at an early stage, and it represents one of the most promising challenges in high-entropy engineering of materials.^57–59^ Assessing such relations would be of utmost importance not only for further enhancing the mechanical and thermal properties of HEAs but also to tailor the microstructure of HEA superconductors by means of improved percolative networks and by tuning the mutual interaction between the short-range configurations. Therefore, on HEA-type BiS_2_-based superconductors and, in general, on HEA-type layered superconductors, a systematic study of the electronic and structural properties is needed to address this significant issue and to gain a novel understanding of the interlayer interactions between the HEA layer and the superconducting one.

In summary, this study has unveiled the intrinsic electronic structure and inhomogeneity present in HEA-type layered REOBiS_2_ superconductors. The intrinsic electronic structure of the HEA-type system is found to be similar to the pristine system, suggesting the robustness of the fundamental electronic structure, even in the presence of large configurational disorder due to mixing entropy. By employing SPEM and space-resolved ARPES, the coexistence of distinct electronic phases has been revealed, each characterized by different density of states at the Fermi level and spatial separation patterns at the submicroscale to mesoscale. These findings point to the crucial role of interplay between the local configuration of rare-earth elements and the distortions of the conducting BiS_2_ layer in modulating the charge transfer mechanisms that drive the emergence of electronic domains. The results suggest that different local arrangements of the HEA layer driven by different substitutions may facilitate a complex interplay of self-doping, structural adjustments, and phase percolation that ultimately shape the material’s superconducting behavior. Space-resolved diffraction and X-ray absorption studies should be useful to make a direct correlation between the electronic and the local structural domain sizes with the mixing entropy. Nevertheless, this work not only advances our understanding of the fundamental electronic properties of high-entropy layered superconductors but also paves the way for targeted approaches to enhance superconducting performance by fine-tuning configurational entropy and short-range atomic order.

## Experimental Section

Several single crystal samples of high-entropy REOBiS_2_ superconductors were synthesized following the growth procedure reported elsewhere.^41,56^ The crystals were characterized for their phase purity, structure, and transport properties prior to the spectroscopy measurements. Scanning electron microscopy (SEM) and energy-dispersive X-ray spectrometry (EDS) were used to characterize homogeneous elemental distribution in the single crystal samples.^41^ Well characterized single crystals with nominal compositions of Ce_0.33_Pr_0.33_Nd_0.33_OBiS_2_ (RE3e) and La_0.20_Ce_0.20_ Pr_0.20_Nd_0.20_Sm_0.20_OBiS_2_ (RE5e) were selected for the present work. The actual chemical compositions, measured by SEM-EDS, were very similar to the nominal ones (Ce_0.33_Pr_0.33_Nd_0.33_OBiS_2.12_ and La_0.23_Ce_0.21_ Pr_0.19_Nd_0.19_Sm_0.17_OBiS_2_).^56^ The two samples are characterized by the mixing entropy Δ*S*_mix_ = 1.098 and 1.598 R, respectively. The samples show zero resistivity below the transition temperature *T*_c_, found to be 3.0 and 2.2 K, respectively, in the temperature-dependent measurements, indicating the bulk nature of the superconducting behavior.^56^

Space-resolved ARPES measurements were performed at the Spectromicroscopy beamline of the Elettra Synchrotron Radiation Facility in Trieste, Italy.^60,61^ For the present measurements, linearly polarized light of energy *hν* = 27 eV was used, focused using a Schwarzschild optics down to a 500 × 500 nm^2^ beam spot. Fermi surface mapping was carried out by changing the position of the electron energy analyzer with the photon beam and fixed sample position. The single crystals were cleaved in situ at 100 K in ultrahigh vacuum (<10^–10^ mbar) to obtain a clean surface. For the present measurements, the total energy resolution was approximately 50 meV, while the angle resolution was limited to 1°. All measurements were made within 12 h after the cleavage, and the sample temperature was kept constant at *T* = 100 K.
